# Exergoeconomic Analysis of Corn Drying in a Novel Industrial Drying System

**DOI:** 10.3390/e22060689

**Published:** 2020-06-20

**Authors:** Bin Li, Chengjie Li, Junying Huang, Changyou Li

**Affiliations:** College of Engineering, South China Agricultural University, Guangzhou 510642, China; libin@stu.scau.edu.cn (B.L.); lichengjie@stu.scau.edu.cn (C.L.); huangjunying@stu.scau.edu.cn (J.H.)

**Keywords:** exergoeconomic, exergy, industrial drying, corn, water

## Abstract

The improvement of the design and operation of energy conversion systems is a theme of global concern. As an energy intensive operation, industrial agricultural product drying has also attracted significant attention in recent years. Taking a novel industrial corn drying system with drying capacity of 5.5 t/h as a study case, based on existing exergoeconomic and exergetic analysis methodology, the present work investigated the exergetic and economic performance of the drying system and identified its energy use deficiencies. The results showed that the average drying rate for corn drying in the system is 1.98 g_water_/g_dry matter_ h. The average exergy rate for dehydrating the moisture from the corn kernel is 345.22 kW and the exergy efficiency of the drying chamber ranges from 14.81% to 40.10%. The average cost of producing 1 GJ exergy for removing water from wet corn kernels is USD 25.971, while the average cost of removing 1 kg water is USD 0.159. These results might help to further understand the drying process from the exergoeconomic perspective and aid formulation of a scientific index for agricultural product industrial drying. Additionally, the results also indicated that, from an energy perspective, the combustion chamber should be firstly optimized, while the drying chamber should be given priority from the exergoeconomics perspective. The main results would be helpful for further optimizing the drying process from both energetic and economic perspectives and provide new thinking about agricultural product industrial drying from the perspective of exergoeconomics.

## 1. Introduction

Drying is the process of removing moisture from natural products (e.g., agricultural products, wood and fruit) or industrial materials (e.g., lignite, ceramics and medical materials) down to a specific moisture content, while ensuring prime product quality, high throughput and minimal operational costs [1]. Drying is a highly energy-intensive operation in grain industrial production. According to the literature [2,3,4], drying operations consume about 10–25% of national energy use each year. Considering the environmental effect and the limited amount of natural resources to produce energy, it is of great economic value and social significance to explore evolutionary and revolutionary technological drying technologies and processes.

In recent decades, researchers have undertaken a large number of studies of new drying technologies and processes [5,6,7,8,9]. Although the existing literature has reported new drying technologies and processes for specific materials, few works have reported on industrial-scale drying systems, especially for grain drying. An industrial drying system is a complex system composed of several parts. In the drying process, different kinds of energy resources (e.g., natural gas, coal) provide the corresponding energy for maintaining the operation of the drying system. Inherent to this process, energy is wasted in the various devices involved due to the irreversibility of energy conversion [10]. It is thus necessary to reveal where and how much energy is lost and destroyed in the drying system. Exergy analysis is an effective method to identify methods and possible benefits of designing more efficient thermal systems through the reduction of existing inefficiencies [11]. Moreover, exergy analysis is an effective tool to evaluate the sustainability and environmental impact of a production system. In 2001, Rosen and Dincer proposed an interdisciplinary triangle for exergy analysis, noting that exergy is the confluence of energy, the environment and sustainable development [12]. In the same year, Rosen and Dincer also illustrated the relationships among environmental impact, sustainability and exergy efficiency. Based on the concept of exergy and its extensions, a significant amount of research has been undertaken on the analysis of the energy utilization level of agricultural product drying systems, as tabulated in Table 1.

As mentioned above, the ultimate objective of drying is to obtain a high-quality dried product with minimal operational costs and maximum benefits. Hence, in addition to energetic performance and quality evaluation, economic evaluations should also be performed of drying systems for specific materials. Exergoeconomic analysis is an interdisciplinary subject which combines exergy analysis and economics analysis organically. The method, which is based on the second law of thermodynamics, introduces the basic ideas of system engineering, optimization theory, and decision theory, and has special advantages in analyzing and optimizing complex energy systems [20]. With the development of exergoeconomics, Lozano, M. A.; and Valero, A. proposed the exergy cost theory [21], which formulates the fundamentals and criteria that enable the description of the cost formation process and the assessment of the efficiency in energy systems. The methodology has been widely verified to be applicable to energy and economic analysis, including of bituminous coal fired power plants [22], drying systems [23] and municipal solid waste digestion plants [24]. Although a large number of reports based on the exergy cost theory have been published in recent years, few works have been reported on the application of the theory to industrial-scale agricultural product drying systems [25,26,27].

In order to achieve the aim of efficient, economical and environmentally-friendly post-harvest processing of grain, researchers have made significant efforts to develop innovative drying systems. For example, Sarker M. S. H. et al., reported an industrial fluidized bed dryer with drying capacity of 22 t/h in 2015. Results of analysis showed that the energy efficiency of the drying process ranged from 5.24% to 13.92%, and the study recommended that energy efficiency should be further improved by recycling the waste energy in the exhaust air and enhancing the insulation of the dryer body [28]. Ma X. Z. et al., introduced a grain counter-flow drying system with drying capacity of 12.5 t/h; the authors found that the heat loss in outlet air ranges from 1.86% to 21.26% of the total heat supply and exergy efficiency should be improved by recycling the waste heat in flue gases [29]. Considering these recommendations and based on our previous work, the present study proposes a novel industrial-scale drying system with a waste heat recovery function developed by our team. The existing advanced energy–exergy methodology was employed to estimate the energetic and exergetic performance and heat recovery behavior of the drying system, while an exergoeconomic methodology was adopted to reveal the costs related to each exergy stream and each component of the complex drying system. In addition, quality aspects, including impurities and the damage ratio of the product, were also investigated to increase the economic benefit in the corn processing industry.

## 2. Materials and Methods

The corn (Variety: Changcheng 799#) was freshly harvested from a local farm at Xinzhou City, Shanxi Province, China. The average initial moisture content of the corn kernel was ascertained to be 32.2 %d.b. using the 105 °C constant weight methodology [30], and the final moisture content of the dried product was considered to be 14 %d.b. [31].

### 2.1. System Description and the Working Principle

**Figure 1:**
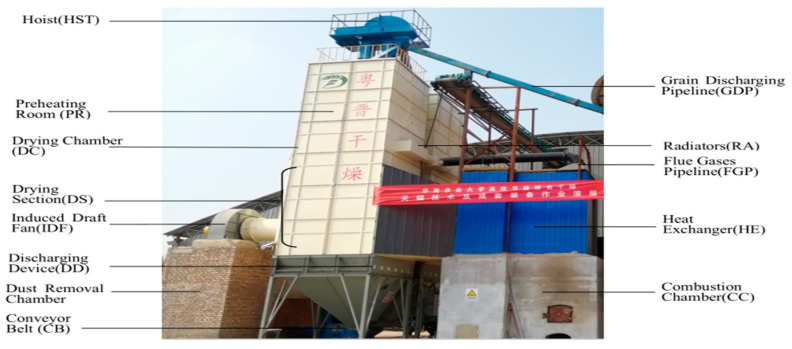
Picture of the industrial drying system.

**Figure 2:**
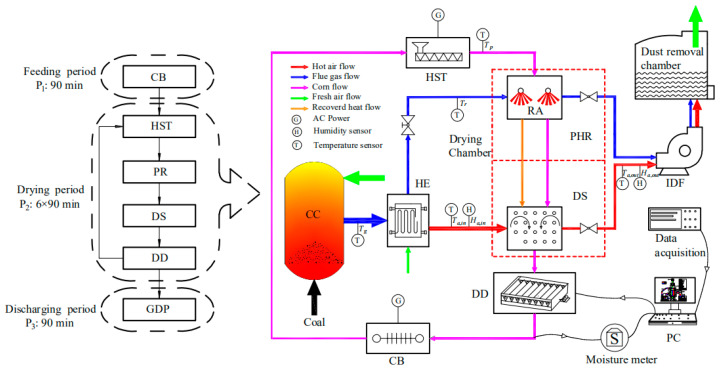
Schematic diagram of the drying system.

The industrial drying system with drying capacity of 5 t/h is shown in Figure 1. As can be clearly seen from the figure, the system consists of eight main components: combustion chamber (CC), heat exchanger (HE), hoist (HST), preheating room (PR) consisting of eight far infrared radiators, drying chamber (DC), induced draft fan (IDF), discharging device (DD), and dust removal chamber. The overall drying operation consists of three periods: Feeding Period (P_1_)—the corn is lifted by hoist and the drying chamber is completely full after 90 min. Drying Period (P_2_)—after the drying chamber is full, the pre-combustible drying chamber, induced draft fan and discharging device are sequentially opened and the whole system then runs for about 9 h. Discharging Period (P_3_)—when the moisture content is about 15 %d.b., the induced draft fan in the drying chamber is shut down, the grain discharge valve on the top of the dryer is opened, and the dried corn is discharged through the grain discharging pipeline, as shown in Figure 1. This period lasts about 90 min. The drying process and the time needed for each period are shown in the Figure 2. The operating data of the system is shown in Table 2.

### 2.2. Data Collection

During the overall drying operation, the temperatures of the drying chamber (*T_DC_*), inlet flue gases (*T_g,in_*), outlet flue gases (*T_g,out_*), outlet corn (*T_c,out_*), inlet air flux (*T_a,in_*), outlet air flux (*T_a,out_*), ambient air (*T*_0_) and radiators (*T_r_*) were measured by temperature sensors inserted into the corresponding components. The humidity of the inlet air flux (*H_a,in_*), outlet air flux (*H_a,out_*) and ambient air (*H*_0_) were measured by corresponding humidity sensors. The measured data were collected by a self-developed data acquisition system. Moreover, the moisture content of the outlet corn (*MC*) was measured at 90-min intervals (the design time for cycling the full dryer is 90 min) using the 105 °C constant weight methodology [27]. Details of the measurement instruments are shown in Table 3.

### 2.3. Drying Kinetics

In this work, the dry basis moisture content (*MC*) and the drying rate (*DR*) were adopted to investigate the drying kinetics of industrial corn drying, and can be calculated according to Equations (1) and (2) [32]:

(1) $$MC=\frac{m_{t}-m_{d}}{m_{d}}\times 100\%$$

(2) $$DR=\frac{MC_{t+\Delta t}-MC_{t}}{\Delta t}\times 100\%$$

where *m_t_* is the weight of the material at time *t,* g; *m_d_* is the weight of absolute dry matter determined using the 105 °C constant weight methodology.

In addition, the average dehydrated water (*m_dehy_*) from the material in any period Δ*t* can be calculated with Equation (3):

(3) $$m_{dehy}=\frac{m_{d}\cdot \left(MC_{t}-MC_{t+\Delta t}\right)}{\Delta t}$$

### 2.4. Uncertainty Analysis

In the present work, the uncertainties of the obtained data were ascertained by means of the methodology introduced by Holman in 2001 [33]; the equation is shown in Equation (4). The results showed that the uncertainties of the experimental data ranged from 0.6 to 3.3, indicating that the reliability of the data used for calculating the indicators adopted in the present work was good, in addition to confirming reproducibility [34].

(4) $$U={\left[{\left(\frac{\partial F}{\partial z_{1}}u_{1}\right)}^{2}+{\left(\frac{\partial F}{\partial z_{2}}u_{2}\right)}^{2}+\ldots \ldots \ldots +{\left(\frac{\partial F}{\partial z_{i}}u_{i}\right)}^{2}\right]}^{1/2}$$

### 2.5. Theoretical Consideration

To investigate the drying system, several assumptions were taken into consideration in the present work, as follows:

- The drying system and its main components were considered to be run under a steady-state regime.
- The initial weight of the corn was considered to be 50,000 kg.
- The temperature gradient existing inside a single rice grain was ignored.
- The inertial flow exergy loss of the air in the chamber was ignored.
- The temperature and the relative humidity of the ambient air on the day of the experiment were considered constant.
- The reference state temperature, pressure and relative humidity were considered to be 281.15 K, 101.325 kPa and 85%, respectively.
- The salvage cost was considered to be 10% of the investment cost and the maintenance cost was taken as 2% of the investment cost [23].
- The oxygen (*O_ar_*), moisture (*M_ar_*), and ash (*A_ar_*) content, and low calorific value (*LHV*) of the coal as received were assumed to be 3.19%, 8.0%, 19.02%, and 6700 kcal/kg, respectively [35].

### 2.6. Exergy Analysis

The generally used exergy balance equation [36] was adopted to analyze the exergy rate of each of the components of the drying system, and is expressed as Equation (5):

(5) $$\sum {\dot{Ex}}_{in}-\sum {\dot{Ex}}_{out}=\sum {\dot{Ex}}_{des}$$

The exergy rate for removing moisture from the material was determined with Equations (6) and (7) [37,38]:

(6) $${\dot{Q}}_{\mathrm{dehy}}={\dot{m}}_{\mathrm{dehy}}h_{lh}$$

(7) $${\dot{Ex}}_{\mathrm{dehy}}=\left(1-\frac{T_{0}}{T_{c}}\right){\dot{Q}}_{dehy}$$

The radiant exergy rate (${\dot{Ex}}_{r}$) recovered by the radiators was computed using Equation (8) [39]:

(8) $${\dot{Ex}}_{r}=\sigma A_{r}\varepsilon_{r}T_{r}^{4}\left(1-\frac{T_{0}}{T_{r}}\right)$$

In the present work, the exergy of the product and air were calculated as follows [38]:

(9) $$Ex=\left(h-h_{0}\right)-T_{0}\left(s-s_{0}\right)$$

(10) $$h-h_{0}=c_{p}\left(T-T_{0}\right)$$

(11) $$s-s_{0}=c_{p}\ln\left(\frac{T}{T_{0}}\right)-R\ln\left(\frac{P}{P_{0}}\right)$$

The exergy rates of the steam entering and leaving the drying chamber were computed by considering the chemical and physical exergies as follows [40]:

(12) $$\dot{Ex}=\dot{Ex^{ph}}+\dot{Ex^{ch}}$$

According to the literature [37,38,41], the physical and chemical exergy rates of steam can be calculated using Equations (13) and (14):

(13) $$\dot{Ex_{a}^{ph}}=\dot{m_{a}}\left\{\left(C_{a}+\omega C_{v}\right)\left(T_{a}-T_{0}\right)-T_{0}\left[\left(C_{a}+\omega C_{v}\right)\ln\left(\frac{T_{a}}{T_{0}}\right)-\left(R_{a}+\omega R_{v}\right)\ln\left(\frac{P_{a}}{P_{0}}\right)\right]\right\}$$

(14) $$\dot{Ex_{a}^{ch}}=\dot{m_{a}}\left\{T_{0}\left[\left(R_{a}+\omega R_{v}\right)\ln\left(\frac{1+1.6078\omega_{0}}{1+1.6078\omega }\right)+1.6078\omega R_{a}\ln\left(\frac{\omega }{\omega_{0}}\right)\right]\right\}$$

In Equation (14), the humidity ratio of the air (*ω_a_*) was determined using Equation (15) [40]:

(15) $$\omega_{a}=0.622\frac{\varphi P_{vs,a}}{P_{a}-\varphi P_{vs,a}}$$

The physical exergy rate of the inlet and outlet corn seed was calculated with Equation (16):

(16) $$\dot{Ex_{c}^{ph}}=C_{c}\dot{m_{c}}\left[(T_{c}-T_{0})-T_{0}\ln\left(\frac{T_{c}}{T_{0}}\right)\right]$$

In the present work, coal (anthracite) without preheating was used as the fuel of the combustion chamber, and its chemical exergy in rate form was determined using Equation (17) [42]:

(17) $$\dot{Ex_{coal}^{ch}}=\dot{m_{coal}}\varphi q_{LHV}$$

where *φ* is the chemical exergy factor of solid coal, which was determined by the following [42]:

(18) $$\varphi =1.009+\frac{1.031O_{ar}+0.116M_{ar}}{100-\left(A_{ar}+M_{ar}\right)}$$

Flue gas is a mixture of many chemical components, and its specific heat and exergy depends on the chemical composition of fuels, excess air ratio, and gas temperature. The exergy calculation model developed by C. Coskun et al. was adopted to calculate the exergy of the flue gas [43]:

(19) $${\dot{Ex}}_{fg}=c_{p,fg}\dot{m_{fg}}\left[\left(T_{fg}-T_{0}\right)-T_{0}\left(\ln\frac{T_{fg}}{T_{0}}\right)\right]$$

(20) $$c_{p,fg}=\frac{c_{p,CO_{2}}}{a_{C}+b_{N}+c_{H}+d_{S}}\cdot \frac{m_{tot,steo}}{m_{fg}}+f_{A}$$

(21) $$c_{p,CO_{2}}=0.1874\times 1.000061^{T_{\mathrm{fg}}}\times T_{fg}^{0.2665}$$

As mentioned in the introduction, exergy is the confluence of energy, environment and sustainable development. Exergy efficiency (*η_ex_*), exergetic sustainability index (*SI*) [41] and exergy destruction ratio (*r_D_*) [44] were adopted to evaluate the exergetic performance of the components and the overall system, and were calculated using Equations (22)–(24):

(22) $$\eta_{ex}=\frac{{\dot{Ex}}_{dehy}}{{\dot{Ex}}_{\mathrm{in}}}=\frac{{\dot{Ex}}_{dehy}}{{\dot{Ex}}_{\mathrm{coal}}+P_{IDF}+P_{HST}+P_{CB}+P_{DD}}$$

(23) $$SI=\frac{1}{\left(1-\eta_{ex}\right)}$$

(24) $$r_{D,k}=\frac{{\dot{Ex}}_{D,k}}{{\dot{Ex}}_{D,TOT}}\times 100\%$$

The values of the parameters needed for the calculations mentioned above are shown in Table 4.

### 2.7. Exergoeconomic Analysis

**Figure 3:**
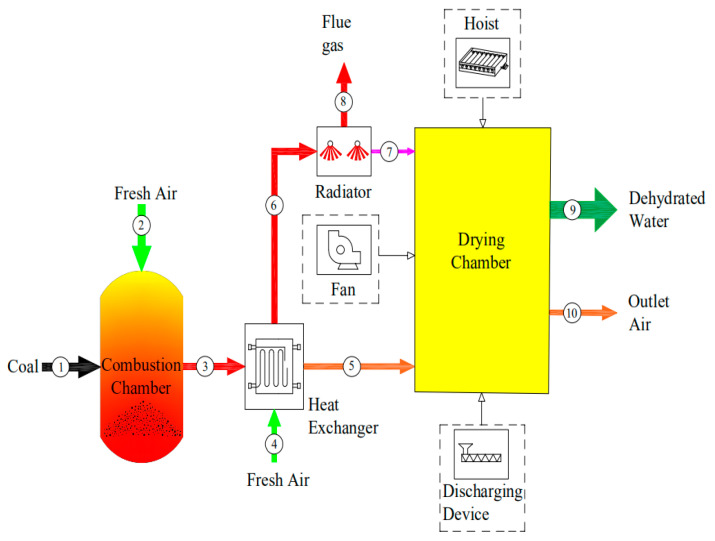
Productive structure of the convective drying system.

One of the most important concepts in exergoeconomic analysis is the “goal”, which is closely related to the reason why the given components are taken into consideration in the design of a system. From an energy perspective, and for a given component or certain process, the fuel is defined to be the amount of exergy provided by the stream inlet into the component, and the product is the exergy provided by the product streams [37]. The ultimate physical goal of the drying is to remove the moisture from the material. Accordingly, as a basic concept of the exergoeconomic analysis, the present work regarded the removed water as the final product of the system, which is affected by multiple exergy flows (chemical exergy, physical exergy and mechanical exergy). The developed productive structure of the drying system is shown in Figure 3, and the exergy balance equations for each component are shown in Table 5.

**Figure 4:**
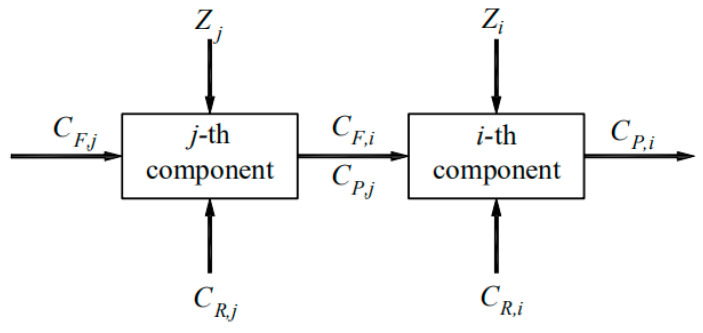
Schematic diagram of the transmission of exergy and cost between any two adjacent subsystems.

Exergoeconomic cost is one of the most important elements of exergoeconomics analysis. For any two adjacent components of the system, the transmission of exergy and cost can be depicted as shown in Figure 4. According to the definition of “product cost = energetic cost + nonenergetic cost” in exergoeconomics analysis [37], the exergoeconomic cost balance equation for a subsystem can be expressed as shown in the figure [48].

(25) $$C_{P,i}=C_{P,j}+C_{R,i}+C_{i}$$

The exergoeconomic cost of each of the flows (*C_i_*) making up the product of the *i*-th component is proportional to its exergy flow, which can be expressed as [27]:

(26) $$C_{i}=Ex_{i}\cdot c_{i}$$

where *c_i_* is the unit exergoeconomic cost of the *i*-th component.

In the present work, the modified productive structure analysis method (MOPSA) introduced by Kim et al. [49] was employed to analyze the exergoeconomic performance of the drying system. Based on the main concept of the methodology, the cost-balance equation for the overall system can be expressed as:

(27) $$c_{9}{\dot{Ex}}_{9}=c_{1}{\dot{Ex}}_{1}+\sum_{i\in \gamma_{n}}Z_{i}$$

One of the advantages of the MOPSA method compared with the traditional specific exergy costing method is that the cost flow rate related to the waste flow streams dissipated at the boundary of a given thermal system can be allocated to each component as the source [50,51]. The cost flow rate of waste of the present system mainly occurs in the flue gas stream at the RA (number 8 in Figure 3) and hot air flux at the DC (number 10 in Figure 3); therefore, the auxiliary equation at the boundary of the overall system can be written as [52]:

(28) $$c_{8}{\dot{Ex}}_{8}+c_{10}{\dot{Ex}}_{10}-c_{R}\sum_{i\in \gamma_{n}}Ex_{D,i}=0$$

Based on the operation schedule shown in Table 2 and the assumptions in Section 2.5, the hourly *Z_ic_*, *Z_sc_* and *Z_mc_* were respectively calculated using Equations (29)–(31) and the results are tabulated in Table 6.

(29) $${\dot{Z}}_{ic}=\frac{Z_{ic}}{2\times 720\times 20}$$

(30) $${\dot{Z}}_{sc}=\frac{Z_{ic}\times 0.1}{2\times 720\times 20}$$

(31) $$\dot{Z_{\mathrm{mc}}}=\frac{Z_{ic}\times 0.02}{2\times 720\times 20}$$

**Figure 5:**
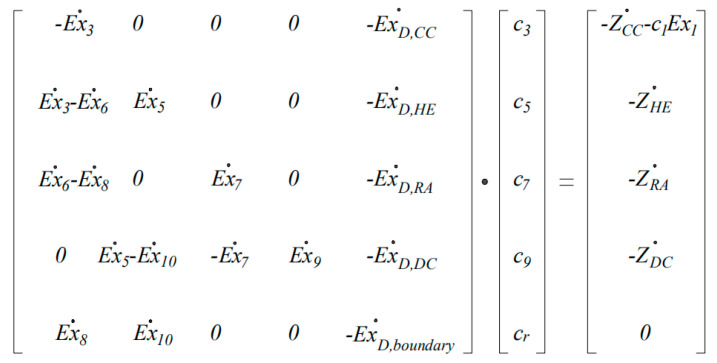
Cost structure of the drying system.

Based on the exergy analysis in Section 2.6 and the exergoeconomic analysis in Section 2.7, the exergoeconomic balance equations of the components are shown in Table 7 and the cost structure of the system is depicted in Figure 5. The relative cost difference (*r_c,i_*) between the product *c_p,i_* and the fuel *c_f,i_*, and the exergoeconomic factor (*f_c,i_*), which indicates the relative contribution of the component-related cost to the sum of costs associated with the *i*-th component [44], were employed to evaluate the energetic and economic performance of the components, and calculated as follows:

(32) $$r_{c,i}=\frac{c_{P,i}-c_{F,i}}{c_{F,i}}$$

(33) $$f_{c,i}=\frac{{\dot{Z}}_{i}}{{\dot{Z}}_{i}+c_{r}{\dot{Ex}}_{D,i}}$$

## 3. Results and Discussion

The stream type and calculation-related parameters used for analyzing the exergoeconomic performance of the industrial corn drying system are listed in the Table 8. The mass flow rate, temperatures, and pressure of the corresponding stream are the average measured values of the overall drying operation unit, while the specific exergy and exergy rates are the calculated values based on the exergy analysis in Section 2.5.

### 3.1. Drying Kinetics

**Figure 6:**
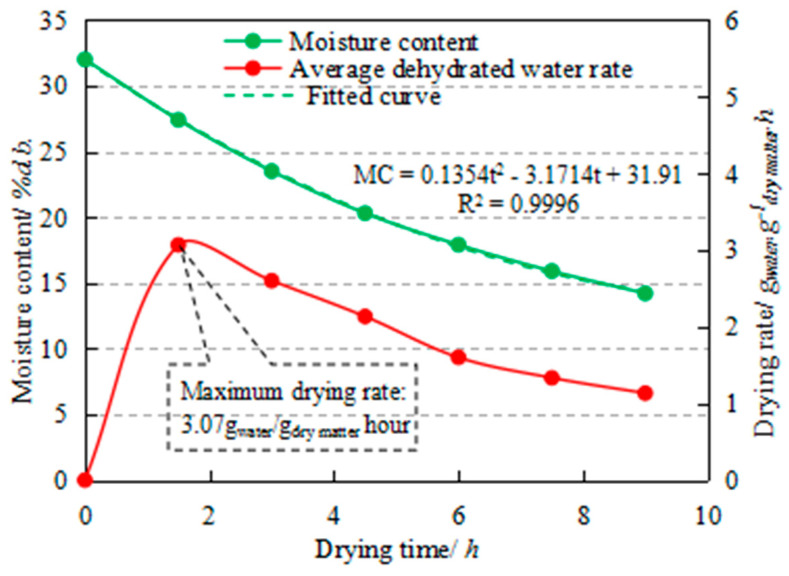
Drying kinetics of the corn industrial drying.

In the present work, the drying kinetics of corn kernels in an industrial dryer with drying capacity of 5.5 t/h were investigated. As mentioned above, the cycling time of the fully loaded dryer was ascertained to be 1.5 h and the moisture content of the corn was measured using the 105 °C constant weight methodology [30] at 90-min intervals. The variations of moisture content and the drying rate with drying time are shown in the Figure 6.

According to the results depicted in Figure 6, the *MC* and *DR* decrease with the increase of drying time, *DR* varied from the minimal 1.13 g_water_/g_dry matter_ h to the maximum 3.07 g_water_/g_dry matter_ h, and the average *DR* was ascertained to be 1.98 g_water_/g_dry matter_ h for the whole drying process. The maximum drying rate (*DR_max_* = 3.07 g_water_/g_dry matter_ h) was found during the first 90 min, which might be because the moisture evaporation of the high moisture content of the material can be considered to be free water evaporation [10]. From the perspective of drying technology, Li CY et al. proposed a variable temperature drying technology that increased the hot air temperature in high moisture content (above 25 %d.b.) to improve the drying rate at a reasonable level of energy consumption for paddy drying [38]. Similar drying technology might be adopted to maximize the drying rate and optimize the energy consumption of corn drying in future work. Experimental data were also simulated and the relationship between *MC* and *t* was found to be *MC* = 0.14*t*^2^ − 3.17*t* + 31.91 (*R*^2^ = 0.9996), which could help predict the moisture content of corn in the drying process.

### 3.2. Exergetic Performance

To identify inefficient energy-consumption components of the drying system and further improve the exergy efficiency of the drying process, the exergetic performance of the components for the overall drying system and the exergetic performance of the drying chamber during the drying process were investigated by applying *SI* and *r_D_*, which respectively reflect the influence of exergy efficiency change on sustainability and the contribution of component-related exergy destruction to overall exergy destruction [53]. The results are shown in Table 9.

As can be seen from the table, for a total drying operation (9 h), the sustainability indexes of the RA (1.15) and DC (1.35) are lower than 2 [14], while those of CC and HE have high values of 2.40 and 5.64, respectively, which indicates that the RA and DC should be improved while HE and CC show a good exergetic performance. For the latter, it is difficult to improve the exergy efficiency of the HE (82.28%) while there is substantial improvement potential for the CC owing to the largest exergy destruction rate, for example, by enhancing the insulation of the combustion chamber wall. Considering the main purpose of the RA is to recycle the waste energy in flue gas, and the *r_D_* of the RA achieved the lowest value (1.83%) among the four components, the improvement priority of the RA is ranked last, even if its *SI* value is the lowest. The exergy destruction ratio analysis shows that the contributors to the total exergy destruction, in ascending order of importance, are as follows: RA, HE, DC and CC. Thus, efforts should firstly be made to improve the CC, followed by the DC, HE and RA.

**Figure 7:**
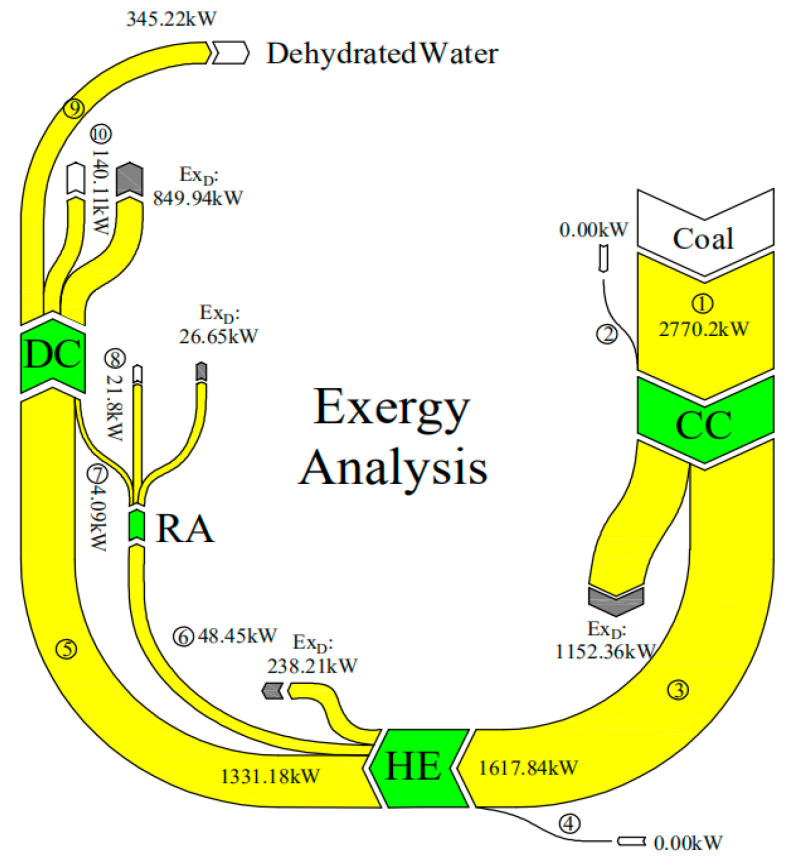
Sankey diagram for the exergy analysis of the overall drying system.

The exergy carried by the fresh air flux was considered to be zero in the present work. Figure 7 depicts the exergy flux among the four main components of the whole drying system. As can be seen from the figure, the initial exergy input rate into the CC is 2770.2 kW. Moreover, there is a significant exergy destruction rate in the CC (1152.36 kW), which indicates that the CC can be greatly improved by reducing its exergy destruction. Such attempts could relate to the fuel types (e.g., natural gas, biomass fuel), insulation of the combustion chamber wall, and physical structure of the drying chamber to improve exergy efficiency, as recommended by Yuanyuan Zhang et al. [54]. For the HE, the fuel is the input flue gas (③ in Figure 7) and the products are the hot air (⑤) and flue gas (⑥). Only 238.21 kW exergy is destroyed, and there is an exergy flow of 48.45 kW into the RA, which respectively account for 14.72% and 2.99% of the total input exergy rate, indicating the HE has a good exergetic performance. More details about the self-developed heat exchanger can be found in the patent (CN104482751A) [55]. Although only 4.09 kW of radiant exergy is recycled by the RA, the improvement of the seed tissue function caused by the far-infrared wavelength cannot be ignored. As reported by Zhu Wenxue et al., corn grains have the highest absorption rate of far-infrared radiation when the far-infrared wavelength is near 9 μm [56]. The average exergy rate for dehydrating moisture from corn kernels was found to be 345.22 kW. Furthermore, there was a high exergy destruction rate in the DC, with the average exergy efficiency ascertained to be 25.85%, indicating the exergy efficiency can be greatly improved.

**Figure 8:**
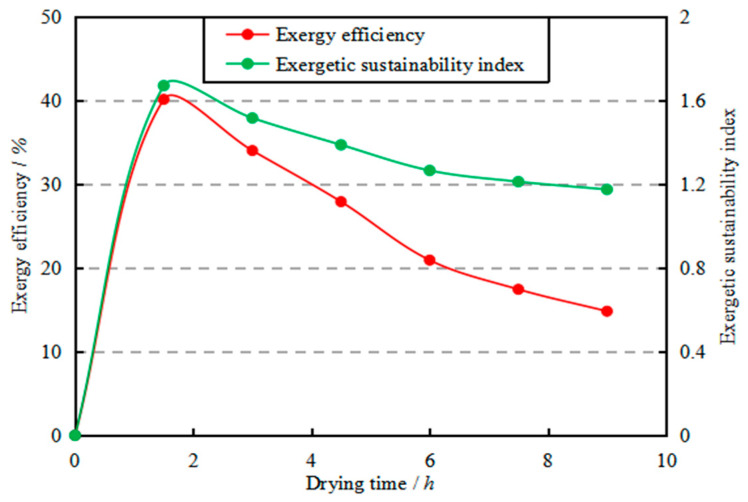
Variations of exergy efficiency and sustainability with drying time.

In order to fully investigate the exergetic performance of the DC, the variation of *η_ex,DC_* with drying time was investigated; results are shown in Figure 8. The figure clearly shows that *η_ex,DC_* and *SI* decrease with the increase of drying time, and respectively range from 14.81% to 40.10% and 1.17 to 1.67. These results are close to those in similar agricultural product industrial dryers, such as an industrial tray dryer for cassava starch drying (16.04% ≤ *η_ex_* ≤ 30.65%; 1.19 ≤ *SI* ≤ 1.44) [57] and a semi-industrial continuous band microwave dryer for paddy drying (4.13% ≤ *η_ex_* ≤ 13.88%; 1.04 ≤ *SI* ≤ 1.16) [49]. After six hours, *η_ex,DC_* was lower than 20%, indicating that attention should be paid to the optimization of the drying kinetic in this period. A lower drying temperature (≤39 °C) might improve the exergy efficiency for a reasonable energy usage, as recommended by M.S.H. Sarker et al. [28].

### 3.3. Exergoeconomic Performance

Based on the exergoeconomic analysis in Section 2.7, the non-energy cost ${\dot{C}}_{i}$ and the exergetic cost of the fuel (${\dot{c}}_{F,I}$) and the product (${\dot{c}}_{P,i}$) for the *i*-th component were investigated. The relative cost difference (*r_c,i_*) and the exergoeconomic factor (*f_c,i_*) were also employed to evaluate the exergoeconomic performance of the system. The results are shown in Table 10.

The non-energetic costs of the *i*-th component were converted into hourly costs in the present work. As can be seem from Table 10, the main contributor to the total non-energetic costs is the DC, which is because the operating and investment costs of the overall system are focused on the DC (Table 5). It can be seen that *r_c,RA_* obtains the highest value (45.6%) among the four components. According to the literature [58], an exorbitant relative cost difference might be caused by low exergy efficiency or exorbitant non-energetic costs of the component. Therefore, the lowest exergy efficiency (Table 9) of the RA can explain the highest value of *r_c,RA_*, while the exorbitant non-energetic costs of the DC can explain the exorbitant value of *r_c,DC_*. In ascending order, the exergoeconomic factors of each component are ranked CC, HE, RA, and DC, indicating that efforts should primarily be made to reduce the investment cost of the DC while maintaining an appropriate exergy efficiency.

**Figure 9:**
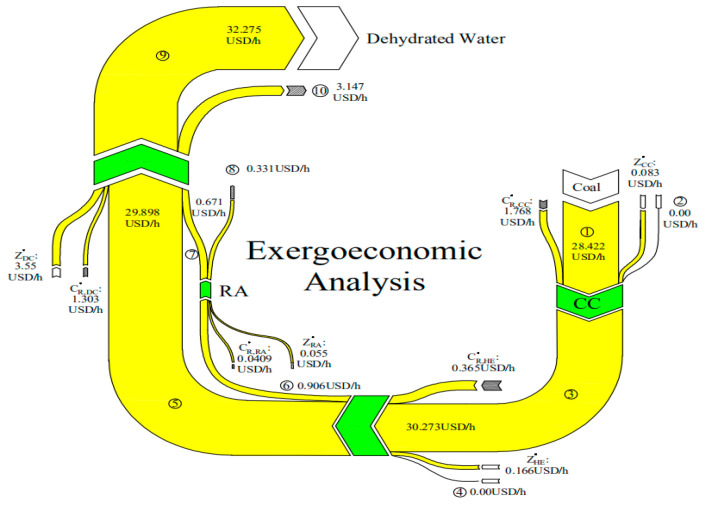
Sankey diagram of the exergoeconomic analysis for the overall drying system.

Based on the arbitrary assumption that the unit exergoeconomic cost of the fresh air stream is equal to zero, the hourly economic costs of all of the streams were calculated; results are depicted in Figure 9. It is clear that the hourly economic cost for dehydrating moisture from corn kernels has a maximum value of 32.275 USD/h. As the final operation unit of the system, *C_ic, DC_* (3.55 USD/h) and *C_R,DC_* (1.303 USD/h) are much higher than those of the other components, which might explain the result. In addition, it is notable that almost 74.06% of the total hourly economic input costs of the RA were recycled, indicating that the RA has a good economic performance. However, based on the exergetic analysis of the RA in Section 3.2, its economic performance can be still improved by reducing the exergy loss in the outlet flue gas or by utilizing material with higher potential IR radiation.

**Figure 10:**
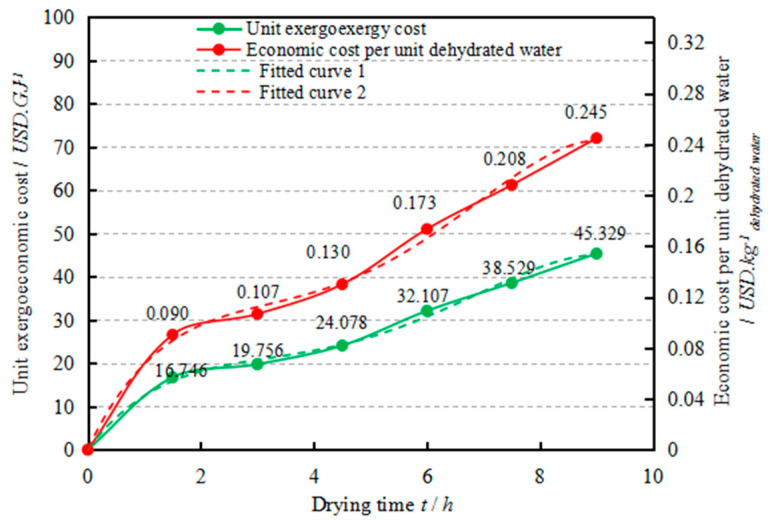
Variation of the unit exergoeconomic cost and economic cost per unit of dehydrated water with drying time for the drying chamber.

In order to comprehensively understand the economic performance of the drying process, the unit exergoeconomic cost (${\dot{c}}_{P,DC}$) and economic cost per unit of dehydrated water (${\dot{c}}_{m}$) were investigated, where ${\dot{c}}_{m}$ was calculated by the equation: ${\dot{c}}_{m}$ = ${\dot{c}}_{P,DC}$·${\dot{E}}_{Xdehy}$/${\dot{m}}_{Xdehy}$. As can be seen from Figure 10, ${\dot{c}}_{P,DC}$ and ${\dot{c}}_{m,DC}$ increase with the increase of drying time, which might due to the fact that more energy is needed to overcome the binding energy between the moisture molecule and the adsorption site in the low moisture content area of the corn kernel [59]. Respectively, ${\dot{c}}_{P,DC}$ and ${\dot{c}}_{m}$ vary from 16.746 to 45.329 USD/GJ and 0.09 to 0.245 USD/kg_dehydrated water_. The drying cost after six hours was twice that of the first three hours, indicating that the drying process should be further optimized (e.g., by utilizing the residual heat of the combustion chamber [60]). Average ${\dot{c}}_{P,DC}$ and ${\dot{c}}_{m}$ were ascertained to be 25.971 USD/GJ and 0.159 USD/kg_dehydrated water_, respectively. Thus, in the presented industrial drying system, the economic cost to produce 1 GJ exergy for removing water is USD 25.971 and the economic cost for removing 1 kg water from wet corn kernels is USD 0.159.

(34) $${\dot{c}}_{{}_{P,DC}}=-0.0002t^{4}+0.0045t^{3}-0.0259t^{2}+0.0912t+0.0012\;(R^{2}=0.9965)$$

(35) $${\dot{c}}_{m}=-0.0409t^{4}+0.8276t^{3}-5.4617t^{2}+16.892t+0.2267\;(R^{2}=0.9965)$$

To simplify the economic cost calculation and enhance the practicability of the exergoeconomic analysis methodology in drying operations, the relationships between ${\dot{c}}_{{}_{P,DC}}$ and ${\dot{c}}_{m}$, and drying time *t*, were established by fitting the corresponding curves (Equations (34) and (35)). These relationships could help in formulating an efficient drying process and predicting the associated drying cost.

## 4. Conclusions

The present work considers dehydrated water as the ultimate productive goal of an industrial drying system. The exergetic and exergoeconomic performances of the drying system were comprehensively investigated. Based on the results achieved, the following conclusions can be drawn:

(1) The drying rate of corn kernels decreases with the increase of drying time, and the average drying rate for corn kernels in the system was ascertained to be 1.98 g_water_/g_dry matter_ h.
(2) The contributors to the total exergy destruction are as follows in ascending order of importance: RA, HE, DC and CC. This indicates that efforts should firstly be made to improve the CC, followed by the DC, HE and RA.
(3) The average exergy rate for dehydrating moisture from corn kernels was found to be 345.22 kW and the exergy efficiency of the drying chamber ranges from 14.81% to 40.10%.
(4) The drying chamber should be firstly optimized because it has the highest exergoeconomic factor of 73.14%. Efforts should also be made to reduce the investment cost of the drying chamber and improve its exergy efficiency.
(5) The average cost of producing 1 GJ exergy for removing water from wet corn kernels is USD 25.971, and the average cost of removing 1 kg water is USD 0.159.

The present work revealed the existing energy use deficiencies in the drying system. Thus, the main results would be helpful for further optimizing the drying process from both energetic and economic perspectives, and also indicate possible areas for enhancing the energy utilization level. Further study is recommended to identify the appropriate drying temperature and air flows for faster drying of corn kernels, to achieve better quality corn at a reasonable economic cost. Furthermore, the environmental impact of the drying operation should also be studied.

## Figures and Tables

**Table 1:** Recent works on energy and exergy analyzes for agricultural product drying systems.

Agro-Product	Drying System	Main Conclusions	References
Cassava starch	Tray dryer	Exergy inflow, exergy outflow and exergy loss increased with increase in both drying air temperature and energy utilization.	[13]
Rough rice	Convective dryer	Exergy efficiencies of the drying process and chamber are in the ranges of 5.10% and 29.41%, and 32.64–67.75%, respectively.	[14]
Onion	Batch dryer	The maximum exergy efficiency is 75.2% while the minimum exergy efficiency is 36.5%.	[15]
Soybeans	microwave-assisted fluidized bed dryer	The microwave power could enhance the thermodynamic efficiency of fluidized bed dryers.	[16]
Kiwi	Microwave drying	Energy and exergy efficiency increased with increasing microwave power and decreasing slice thickness while values of energy efficiency (15.15–32.27%) were higher than exergy efficiency (11.35–24.68%).	[17]
Tomato slices	Heat pump dryer	The highest mean specific moisture extraction ratio and coefficient of performance of heat pump drying system are 0.324 kg/kWh and 2.71, respectively.	[18]
Grains and Fenugreek seeds	Wall heated fluidized bed dryer	The energy utilization ratio increased with increasing wall temperature, air velocity, bed height and initial moisture content and decreased with drying time.	[19]

**Table 2:** Data on operating period.

Item	Values
Corn drying month for a year (month/year)	2
Duration of drying period (hour/month)	720
Economic life (year)	20
Local market price of anthracites (USD/t)	85.3
Electricity price for industrial production (USD/kWh)	0.105

**Table 3:** Details of the experimental instruments.

Devices	Model	Measurement Range	Precision
Thermal resistance	PT100	−200–450 °C	±0.1 °C
Thermocouple	WRN-130/230	0–1300 °C	±0.1 °C
Anemometer	DT-8893	0.001–45 m/s	0.01 m/s
Temperature and humidity sensors	AM2301	0–100%/−40–80 °C	±3%/±0.5 °C
Data acquisition system	Self-developed	-	-

**Table 4:** The values of the parameters adopted in the present work.

Parameter Name	Value/Equation	Unit	Reference
*R_a_*	0.287	kJ·kg^−1^·K^−1^	[45]
*R_v_*	0.462	kJ·mol^−1^·K^−1^	
*σ*	5.67 × 10^−8^	W·m^–2^·K^−4^	[39]
*ε_r_*	0.9	-	
*A_r_*	16.6	m^2^	
$\dot{m}$ *_p_*	9.26	kg·s^−1^	
*h_lh_*	$h_{\mathrm{lh}}=2.503\times 10^{6}-2.386\times 10^{3}{\left(T-273.16\right)}^{0.5}{{}_{{}_{}}}_{}\;273.16\leq T\leq 338.72$	kJ·kg^−1^	[46]
*C_v_*	$\begin{array}{l} C_{v}=1.883-\left(1.6737\times 10^{-4}T\right)+\left(8.4386\times 10^{-7}T^{2}\right)\\ -\left(2.6966\times 10^{-10}T^{3}\right) \end{array}$	kJ·kg^−1^·K^−1^	[47]
*C_a_*	$\begin{array}{l} C_{a}=1.04841-\left(3.83719\times 10^{-4}T\right)+\left(9.45378\times 10^{-7}T^{2}\right)\\ -\left(5.49031\times 10^{-10}T^{3}\right)+\left(7.9298\times 10^{-14}T^{4}\right) \end{array}$	kJ·kg^−1^·K^−1^	
Radiator size	D_0_ = 0.22; D_i_ = 0.2; L = 3	m	

**Table 5:** Fuel exergy, product exergy, exergy dissipation and exergy efficiency of the components of the system.

Component	Fuel Exergy	Product Exergy	Exergy Dissipation	Exergy Efficiency
CC	${\dot{Ex}}_{1}+{\dot{Ex}}_{2}$	${\dot{Ex}}_{3}$	${\dot{Ex}}_{1}+{\dot{Ex}}_{2}-{\dot{Ex}}_{3}$	$\eta_{ex,CC}={\dot{Ex}}_{3}/({\dot{Ex}}_{1}+{\dot{Ex}}_{2})$
HE	${\dot{Ex}}_{3}+{\dot{Ex}}_{4}$	${\dot{Ex}}_{5}$	${\dot{Ex}}_{3}+{\dot{Ex}}_{4}-{\dot{Ex}}_{5}-{\dot{Ex}}_{6}$	$\eta_{ex,HE}={\dot{Ex}}_{5}/{\dot{(Ex}}_{3}+{\dot{Ex}}_{4}-{\dot{Ex}}_{6})$
RA	${\dot{Ex}}_{6}$	${\dot{Ex}}_{7}$	${\dot{Ex}}_{6}-{\dot{Ex}}_{7}-{\dot{Ex}}_{8}$	$\eta_{ex,RA}={\dot{Ex}}_{7}/{\dot{(Ex}}_{6}-{\dot{Ex}}_{8})$
DC	${\dot{Ex}}_{5}+{\dot{Ex}}_{7}$	${\dot{Ex}}_{9}$	${\dot{Ex}}_{5}+{\dot{Ex}}_{7}-{\dot{Ex}}_{9}-{\dot{Ex}}_{10}$	$\eta_{ex,DC}={\dot{Ex}}_{9}/({\dot{Ex}}_{5}+{\dot{Ex}}_{7}-{\dot{Ex}}_{10})$

**Table 6:** Non-energetic costs of the subsystems.

Subsystem	*Z_ic_* (USD/h)	*Z_sc_* (USD/h)	*Z_mc_* (USD/h)	Total Non-Energy Cost (USD/h)
CC	7.41 × 10^−2^	7.41 × 10^−3^	1.48 × 10^−3^	8.30 × 10^−2^
HE	1.48 × 10^−1^	1.48 × 10^−2^	2.96 × 10^−3^	1.66 × 10^−1^
RA	4.94 × 10^−2^	4.94 × 10^−3^	9.88 × 10^−4^	5.53 × 10^−2^
DC	3.17	3.17 × 10^−1^	6.34 × 10^−2^	3.55
Whole system	3.44	3.44 × 10^−1^	6.88 × 10^−2^	3.85
Real time exchange rate: 1 USD = 7.0308 CNY				

**Table 7:** Cost balance equations, F-rules [44] and arbitrary assumptions computed for all of the components of the drying system.

Component	Cost Balance	Unit Exergoeconomic Cost
CC	${\dot{c_{1}Ex}}_{1}+c_{2}{\dot{Ex}}_{2}-c_{3}{\dot{Ex}}_{3}-c_{r}{\dot{Ex}}_{D,CC}+\dot{Z_{CC}}=0$	*c*_1_ = 2.85 *USD/GJ*; *c*_2_ = 0 (arbitrary assumption)
HE	$c_{3}{\dot{Ex}}_{3}+c_{4}{\dot{Ex}}_{4}-c_{6}{\dot{Ex}}_{6}-c_{5}{\dot{Ex}}_{5}-c_{r}{\dot{Ex}}_{D,HE}+\dot{Z_{HE}}=0$	*c*_3_ = *c*_6_ (F-rule); *c*_4_ = 0 (arbitrary assumption)
RA	$c_{6}{\dot{Ex}}_{6}-c_{8}{\dot{Ex}}_{8}-c_{7}{\dot{Ex}}_{7}-c_{r}{\dot{Ex}}_{D,RA}+\dot{Z_{RA}}=0$	*c*_6_ = *c*_8_ (F-rule)
DC	$c_{5}{\dot{Ex}}_{5}+c_{7}{\dot{Ex}}_{7}-c_{10}{\dot{Ex}}_{10}-c_{9}{\dot{Ex}}_{9}-c_{r}{\dot{Ex}}_{D,DC}+\dot{Z_{DC}}=0$	*c*_5_*= c*_10_ (F-rule)*; c*_9_ (final product exergy cost)

**Table 8:** The stream type, physical parameters, and corresponding exergy rate and specific exergy used for analyzing the exergoeconomic performance of the drying system for the overall drying process.

No.	Stream Type	Temperature (K)	Pressure (bar)	Mass Flow Rate (kg·s^−1^)	Enthalpy Rate (kW)	Entropy J/kg.K	Exergy Rate (kW)	Specific Exergy (kJ·kg^−1^)
1	Coal	281.15	-	0.093	-	-	2770.20	29,918.11
2	Fresh air	281.15	1.01	1.74	0	0	0.00	0.00
3	Flue gas	1056.34	3.42	1.78	2382.17	1326.94	1617.84	908.90
4	Fresh air	281.15	1.01	6.98	0	0	0.00	0.00
5	Hot air	358.75	2.20	6.98	2525.07	22.35	1331.18	195.76
6	Flue gas	388.46	2.62	1.78	644.23	27.64	48.45	27.22
7	Radiation flux	-	-	-	-		4.09	-
8	Flue gas	344.45	1.84	1.78	551.74	10.59	17.71	9.95
9	Dehydrated water	-	-	0.1387	538.38		345.22	2488.37
10	Outlet air	297.25	1.21	6.98	2083.27	4.06	140.1	20.0

Note: density of the fresh air at 8 °C, 1.01 bar was ascertained to be 1.256 kg·m^−3^.

**Table 9:** The exergetic performance of the components for the overall drying system.

Components	${\dot{Ex}}_{in}\;(\mathrm{kW})$	${\dot{Ex}}_{out}\;(\mathrm{kW})$	${\dot{Ex}}_{D}\;(\mathrm{kW})$	*η_ex_* (%)	*SI*	*r_D_* (%)	Improvement Priority
CC	2770.2	1617.84	1152.36	58.41	2.40	47.52	1
HE	1617.84	1379.63	238.21	82.28	5.64	9.82	3
RA	48.45	21.8	26.65	13.08	1.15	1.83	4
DC	1335.27	485.33	849.94	25.85	1.35	40.83	2

**Table 10:** The exergoeconomic performance of the components for the overall drying system.

Components	${\dot{C}}_{i}\;(\mathrm{USD}/h)$	${\dot{c}}_{F,i}\;(\mathrm{USD}/\mathrm{GJ})$	${\dot{c}}_{P,i}\;(\mathrm{USD}/\mathrm{GJ})$	*r_c,i_* (%)	*f_c,i_* (%)	Improvement Priority
CC	8.30 × 10^−2^	2.85	5.20	82.46	4.48	4
HE	1.66 × 10^−1^	5.20	6.24	20	31.24	3
RA	5.53 × 10^−2^	5.20	45.60	776.89	57.49	2
DC	3.55	6.36	25.97	308.33	73.14	1
